# Air Pollution and Acute Myocardial Infarction Hospital Admission in Alberta, Canada: A Three-Step Procedure Case-Crossover Study

**DOI:** 10.1371/journal.pone.0132769

**Published:** 2015-07-13

**Authors:** Xiaoming Wang, Warren Kindzierski, Padma Kaul

**Affiliations:** 1 School of Public Health, University of Alberta, Edmonton, Alberta, Canada; 2 Canadian Vigour Centre, Department of Medicine, University of Alberta, Edmonton, Alberta, Canada; The Ohio State University, UNITED STATES

## Abstract

Adverse associations between air pollution and myocardial infarction (MI) are widely reported in medical literature. However, inconsistency and sensitivity of the findings are still big concerns. An exploratory investigation was undertaken to examine associations between air pollutants and risk of acute MI (AMI) hospitalization in Alberta, Canada. A time stratified case-crossover design was used to assess the transient effect of five air pollutants (carbon monoxide (CO), nitrogen dioxide (NO_2_), nitric oxide (NO), ozone (O_3_) and particulate matter with an aerodynamic diameter ≤2.5 (PM_2.5_)) on the risk of AMI hospitalization over the period 1999–2009. Subgroups were predefined to see if any susceptible group of individuals existed. A three-step procedure, including univariate analysis, multivariate analysis, and bootstrap model averaging, was used. The multivariate analysis was used in an effort to address adjustment uncertainty; whereas the bootstrap technique was used as a way to account for regression model uncertainty. There were 25,894 AMI hospital admissions during the 11-year period. Estimating health effects that are properly adjusted for all possible confounding factors and accounting for model uncertainty are important for making interpretations of air pollution–health effect associations. The most robust findings included: (1) only 1-day lag NO_2_ concentrations (6-, 12- or 24-hour average), but not those of CO, NO, O_3_ or PM_2.5_, were associated with an elevated risk of AMI hospitalization; (2) evidence was suggested for an effect of elevated risk of hospitalization for NSTEMI (Non-ST Segment Elevation Myocardial Infarction), but not for STEMI (ST segment elevation myocardial infarction); and (3) susceptible subgroups included elders (age ≥65) and elders with hypertension. As this was only an exploratory study there is a need to replicate these findings with other methodologies and datasets.

## Introduction

Adverse associations between air pollution and myocardial infarction (MI) are widely reported in medical literature [1–30] and a recent systematic review [31] concluded significant associations with MI short-term risk increase for all pollutants except ozone. While part of the associations are to some extent apparent, mechanisms underlying these associations are not completely understood [20]. Associations of health effects (particularly short-term) with air pollutants are often relatively small [32]; and these types of studies can often have unfavorable signal-to-noise ratios, and substantial correlations among both the exposures and the potential confounders [33]. The health effects can be confounded by study design; lack of sufficient adjustment for covariates; and flexibility in data collection, defining and quantifying exposure, and analysis and reporting [34–36] and lead to variable results.

To highlight this, we summarized case-crossover study designs cited in PubMed before Sep 23 2014 that reported associations between ambient particulate matter (PM) and MI (Table A in S1 File). A number of observations are made from Table A in S1 File: (i) most studies used univariate or simple models adjusted/matched with selected meteorological factors (typically relative humidity and temperature), and only a few studies were adjusted with a second air pollutant; (ii) the findings did not always agree with each other across studies; and (iii) negative/protective effects were not reported. Of eleven studies in Table A in S1 File investigating associations between fine particulate matter (PM_2.5_) and MI, three studies found no associations [1,12,21], one of which included a very large sample of 452,343 MI cases [1]; and eight studies reported associations [3–5,7,15,18,19,23].

We undertook an exploratory study examining associations between air pollutants and acute myocardial infarction (AMI) hospital admission in Alberta, Canada. Of the various ways described above in which these investigations can be confounded, we attempted to address issues of i) adjustment uncertainty or lack of adjustment with co-pollutants commonly present in the atmosphere and other meteorological variables (e.g., wind speed) [33,37], and ii) and regression model uncertainty [38]. For this we developed a three-step procedure—nonparametric univariate (simple model) testing, multivariate logistic regression analysis fully adjusted for co-pollutant and meteorological variables, and bootstrap model averaging. The multivariate analysis was used in an effort to address adjustment uncertainty; whereas the bootstrap technique was used as a mechanism to account for uncertainty in our regression model. We applied this procedure to data in Alberta spanning the period April 1, 1999 to March 31, 2010, searching in a large amount of candidates for potential associations between air pollutants and AMI hospital admission.

## Materials and Methods

### Health Administrative Data

Using Alberta Health administrative databases, a province-provider-registry system in Alberta, we obtained all de-identified historical patient records with a primary diagnosis code of AMI, International Classification of Diseases, version 10 (ICD-10), code I21-I22, or version 9 (ICD-9), code 410. The resulting cohort with 25,894 patients was defined as: patient with his/her first AMI admission event during April 1, 1999 to March 31, 2010; aged 20 or over and resident of Alberta during AMI event period; living 15 km or less to the closest effective air pollution monitoring station in Alberta; and living 50 km or less to the closest effective meteorological monitoring station in Alberta.

We classified a patient’s event with an AMI code as I214 (ICD-10) or 4107 (ICD-9) as having Non-ST Segment Elevation Myocardial Infarction (NSTEMI), and the others as ST Segment Elevation Myocardial Infarction (STEMI). Secondary diagnosis codes (diagnosis 2–25) were used to define comorbidities for each patient, including hypertension (ICD-10 codes I10-I13 and I15, or ICD-9 codes 401), diabetes (ICD-10 codes E10- E14, or ICD-9 codes 250), and dysrhythmia (ICD-10 codes I47-I49, or ICD-9 codes 427). Both primary and secondary diagnosis codes were used for identifying if a patient had prehistory of heart disease (ICD-10 codes I20-I25, or ICD-9 codes 410–414) before his/her AMI hospitalization event.

Sex and age (at the start date of an AMI hospitalization event) were used to define four sub-cohorts (sub-cohorts of Male and Female, and sub-cohorts of Agecat1 and Agecat2 corresponding to patients with age <65 or patients with age ≥65). Patients in the main cohort or in one of the four sub-cohorts were further divided into subgroups defined by AMI type or comorbidities, including: all patients in the cohort or sub-cohort, patients with NSTEMI, patients with STEMI, patients with hypertension, patients with diabetes, patients with dysrhythmia, and patients with a prehistory of heart disease. Table 1 lists sample size for each of these groups.

**Table 1:** Number of hospitalizations for acute myocardial infarction in Alberta subgroups (1999–2009).

**Cohort**	**Whole**	**STEMI**	**NSTEMI**	**Diabetes**	**HTN**	**Dysrhy**	**PIHD**
****MAIN****	25,894	12,750	13,144	6,209	13,733	4,501	7,415
****MALE****	17,488	8,998	8,490	4,011	8,640	2,904	4,892
****FEMALE****	8,406	3,752	4,654	2,198	5,093	1,597	2,523
****AGECAT1****	11,655	6,489	5,166	2,311	5,234	1,120	1,946
****AGECAT2****	14,239	6,261	7,978	3,898	8,499	3,381	5,469

Note: AGECAT1 = age <65; AGECAT2 = age ≥65; STEMI = ST Segment Elevation Myocardial Infarction; NSTEMI = Non-ST Segment Elevation Myocardial Infarction; HTN = Hypertension; Dysrhy = Dysrhythmia; PIHD = prehistory of heart disease.

### Air Pollutant and Meteorological Data

Air pollution data for Alberta were obtained from the Environment Canada National Ambient Pollution Surveillance (NAPS) database [39] for the January 1999 to December 2010 period and linked to patients in the cohort. The NAPS database contains quality assured data compiled by Environment Canada for air monitoring stations across Canada. Station locations in Alberta are shown in Fig A in S1 File (left panel). Hourly records of five criteria air pollutants from a total of 65 monitor stations in Alberta were available during the study period—carbon monoxide (CO, with 14 stations), nitric oxide (NO, with 51 stations), nitrogen dioxide (NO_2_, with 51 stations), ozone (O_3_, with 41 stations), and particulate matter with an aerodynamic diameter ≤2.5 (PM_2.5_, with 40 stations). We were unable use sulfur dioxide (SO_2_) as a covariate because of limited availability of pollutant records. SO_2_ is recognized as an important criteria air pollutant in urban areas and an earlier study reported associations with acute myocardial infarction hospital admissions London [40]. However our initial sample size before linkage with SO_2_ data was 25,895 hospitalizations (Table 1), and after linkage with available SO_2_ data from the NAPS database [39] we only had 18,011 hospitalizations (69.55% of the initial sample). This was too small to permit analysis of subgroups. Our main interest was in exploring the usefulness of the three-step procedure to address adjustment uncertainty and regression model uncertainty. From hourly concentration data for each of the other air pollutants we calculated and used five concentration variables to represent each air pollutant for each day: daily average (i.e., 24-hour average), 6-hour average for the hours 07:00 to 10:00 and 17:00 to 20:00, 12-hour average for the hours 07:00 to 19:00, daily 1-hour maximum and daily 1-hour minimum.

Daily meteorological data were obtained from the United States National Climatic Data Center (NCDC) [41]. NCDC provides historical daily meteorological records for air temperature (daily average, minimum and maximum temperature, in °C), daily average dew point temperature (°C), and daily average wind speed (in meter per hour). Historical records from 209 meteorological monitoring stations in Alberta were available for the study period. Locations of these stations in Alberta are shown in Fig A in S1 File (right panel). We used five variables to represent meteorological data for each day: minimum- and maximum-temperature, apparent-temperature [42], average dew point temperature and average wind speed.

We adopted the following procedure to link patients in the cohort to air pollution data: (1) latitude and longitude of both the postal-codes of patients and NAPS stations were used to calculate a matrix of distance from each postal-code to each station; (2) a list of stations (up to 20) within 15 km was found for each postal-code, ordered from closest to farthest if the list was not null; (3) each patient was linked to a list of stations via postal-code; (4) checking in the list of stations from the first to the last, available NAPS records were found for each patient dated the same month with the exposure date (defined as 0-5^th^ day before the onset day); and (5) patients without NAPS records were eliminated. The linkage procedure was conducted separately for each pollutant because each had a different set of monitoring stations. Patients without records for any one of the five pollutants were eliminated. A similar strategy was used to link patients to meteorological data. After linkage to air pollution and meteorological data, some patients still had a few (or a small fraction) missing records for some air pollutant and meteorological variables. About 3.01% of patients had at least one missing record in the 25 NAPS variables that we used. About 0.01% of patients had at least one missing record in the 5 NCDC meteorological variables that we used. A linear interpolation method was used for imputation of these missing records.

### Ethics Statement

Ethical approval for the study was granted by the University of Alberta’s Health Research Ethics Board-Health Panel (IREB Pro00010852). Patient records/information was anonymized and de-identified with a unique scrambled ID and released to us in this form by the ministry of health prior to analysis.

### Statistical Analysis

We used a case-crossover design [43] with the k^th^ day (k ranging from 0 to 5) before onset of an AMI hospitalization event as the case exposure period for a patient in the cohort. For selection of reference periods using a time-stratified reference-selection design [44,45], the whole study period was stratified into calendar months, and all days in the same year, same month and matching weekday of the hazard exposure day were selected as controls. This strategy is reported as a preferred approach for minimizing confounding by time-trend as well as overlap bias [46].

The following three-step procedure was used for searching for associations. In Step 1 we initially searched a total of 5,250 candidate variables in an univariate analysis—defined by 5 cohorts, 7 subgroups, 5 pollutants, 5 types of daily pollutant concentrations and 6 different lag times (0- to 5-day lag)–for potentially significant associations for AMI hospitalization. Searching was done one-at-a-time using the nonparametric Wilcoxon test and a p-value ≤0.1 as the reference point to identify candidate variables for Step 2.

When estimating health effects from air pollution, it is important to properly adjust for all confounding factors, including exposure to co-pollutants [33,37]. In Step 2 a multivariate conditional logistic regression model was built for each candidate variable of interest. The model was fully adjusted for 20 pollution variables from other pollutants and the 5 metrological variables. A stepwise selection procedure was adopted to eliminate redundant variables and critical level for a variable entry and critical level for a variable stay were both set at 0.25. Coefficient estimation and OR estimation were calculated using the interquartile difference (i.e., difference between the 25th and the 75th percentile concentration) for the candidate variable of interest. Only if a candidate variable had p-value ≤0.05, was it selected into Step 3.

Model uncertainty is an important issue in the interpretation of air pollutant–acute health effect associations [38,47]. An important air pollutant–acute health event association should be replicable for multiple datasets. The bootstrap technique is a computer-intensive resampling method that provides a direct computational way of testing this assumption and assessing model uncertainty by repeated sampling from a set of data [48]. We used the bootstrap technique in Step 3 as a data perturbation method to create a set of 1,000 ‘similar data environments’ and then performed multivariate analysis described in Step 2 1,000 times. Medians from the 1,000 multivariate logistic regression models were used to represent central tendency for statistical parameters of interest. We reported the frequency (number of times) that a candidate variable of interest from Step 2 had p-value ≤0.05 for the 1,000 multivariate logistic regression model replications as a simple measure of how robust/reliable an association is. Like Bayesian model averaging [35], bootstrap model averaging incorporates model uncertainty that results from searching through a set of candidate models and results are obtained easier than through Bayesian analysis [48]. In our case, model averaging was only performed on those variables identified as being potentially important after full adjustment for possible confounders. Further comparative analysis was employed to confirm some of the suggested robust associations found in the three-step procedure. All related analyses were conducted in SAS (release 9.3; SAS Institute, Cary, NC).

## Results

**Fig 1:**
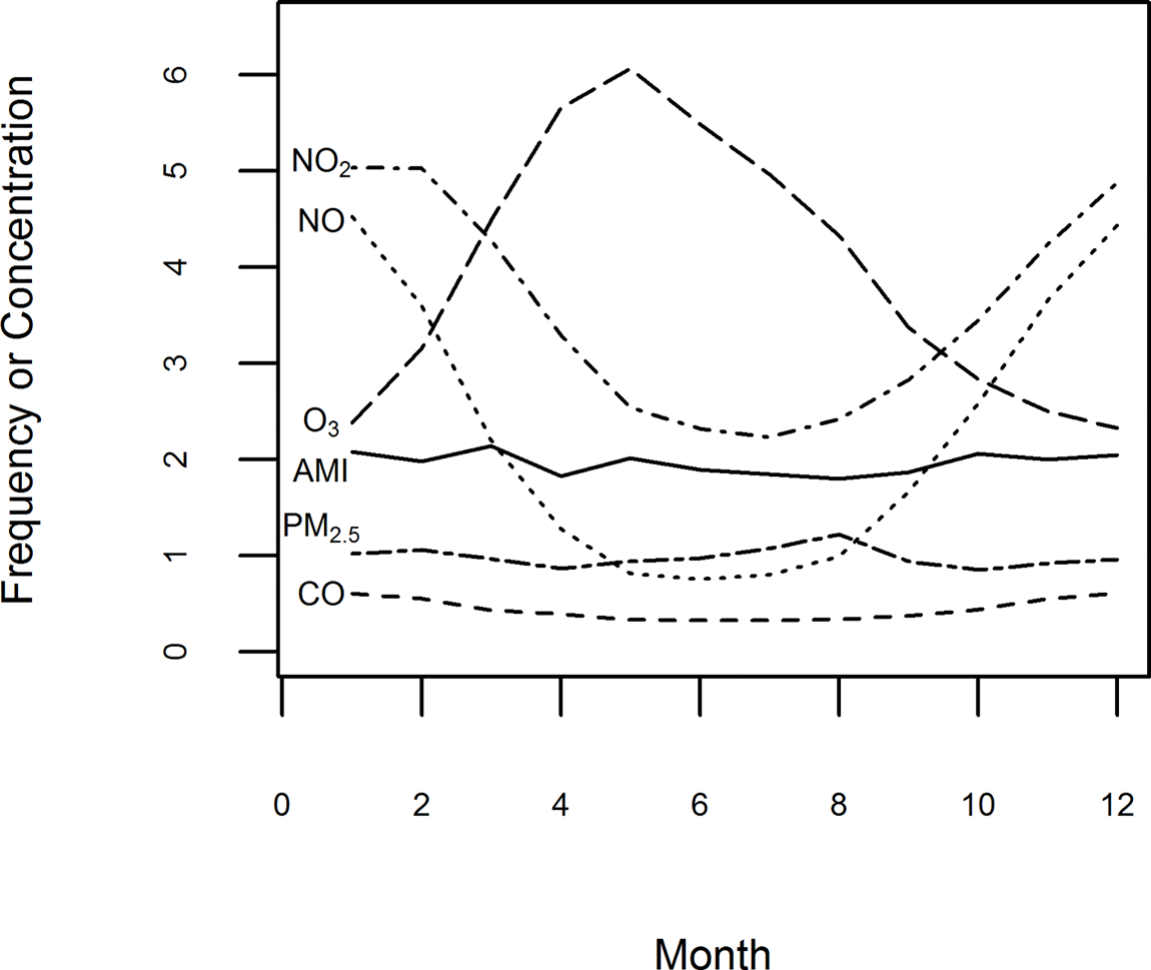
Seasonal trends of monthly average frequency of AMI hospitalizations and monthly average concentrations of air pollutants (April 1999 –March 2010). Monthly frequency of AMI hospitalizations (1 unit = 100) were the average number of events by month. Monthly average concentration levels of CO (1 unit = 1 mg/m^3^), NO (1 unit = 10 μg/m^3^), NO_2_ (1 unit = 10 μg/m^3^), O_3_ (1 unit = 10 μg/m^3^), or PM_2.5_ (1 unit = 10 μg /m^3^) were averaged by month in which the daily mean concentrations linked to the event days.

There were a total 25,894 hospital admission records for AMI (average of 6.45 hospital admission events per day). Fig 1 displays a summary of monthly average frequency of AMI hospitalizations and monthly average levels of the air pollutants at Alberta NAPS air monitoring stations over the study period. Obvious seasonal trends are apparent for several of the air pollutants. Much higher (lower) NO and NO_2_ levels occur during winter (summer) which is opposite to that of O_3_, which has lower (higher) levels occurring during winter (summer). The highest monthly PM_2.5_ levels occur during the summer period (mid-June to mid-September). As indicated in Fig 1, the monthly frequency of AMI hospitalizations averaged over the study period did not differ substantially compared to monthly levels of NO_2_, NO and O_3_ averaged over the study period. While this figure shows that AMI hospitalizations are insensitive to levels of NO_2_, NO and O_3_ averaged over the study period, concentrations used to represent each of these pollutants in the analysis were related to various daily levels (i.e., 24-hour, 6-hour and 12-hour averages, and daily 1-hour maximum and 1-hour minimum levels).

After Step 1 univariate analysis there were 192 of the 5,250 candidate variables with p-values ≤0.1 (Table B in S1 File). After Step 2 multivariate analysis with full adjustment there were only 37 variables with p-value ≤0.05 from the list of 192 (Table 2), reflecting many of the variables exhibiting only weak associations with AMI prior to full adjustment. Results from Step 3 bootstrap model averaging are shown in Table 3. The measure of effect size based on median p-value weakened after bootstrapping for most of the variables (26 of 37) in Table 3. More importantly, 9 of 37 variables no longer had p-value ≤0.05 after bootstrapping; illustrating the importance of controlling for model uncertainty. The frequency that a variable had p-value ≤0.05 from the 1,000 model replications for each of the 37 variables is reported in the last column of Table 3. The lowest frequency was 87 which, although statistically significant, is not at all suggestive of a robust finding.

**Table 2:** Step 2 multivariate analysis with full adjustment results (p-value ≤0.05).

**Cohort**	**Subgroup**	**Variable**	**Lag (day)**	**Estimate**	**StdErr**	**p-value**	**OR**	**Lower CL**	**Upper CL**
****MAIN****	Whole	NO2_AVE12	1	0.0521	0.0173	0.0025	1.054	1.018	1.090
****MAIN****	Whole	NO2_AVE6	1	0.0480	0.0176	0.0063	1.049	1.014	1.086
****MAIN****	Diabetes	O3_MIN	1	0.0347	0.0171	0.0430	1.035	1.001	1.071
****MAIN****	HTN	NO2_AVE	1	0.0793	0.0246	0.0013	1.083	1.032	1.136
****MAIN****	HTN	NO2_AVE12	1	0.0718	0.0241	0.0029	1.074	1.025	1.126
****MAIN****	HTN	NO2_AVE6	1	0.0743	0.0242	0.0022	1.077	1.027	1.129
****MAIN****	Dysrhy	PM25_AVE	0	-0.0466	0.0174	0.0076	0.954	0.922	0.988
****MAIN****	Dysrhy	PM25_AVE12	0	-0.0423	0.0168	0.0119	0.959	0.928	0.991
****MAIN****	Dysrhy	PM25_MAX	0	-0.0343	0.0152	0.0242	0.966	0.938	0.996
****MALE****	Dysrhy	PM25_AVE	0	-0.0590	0.0215	0.0061	0.943	0.904	0.983
****MALE****	Dysrhy	PM25_AVE12	0	-0.0570	0.0209	0.0063	0.945	0.907	0.984
****FEMALE****	Whole	CO_AVE6	1	0.0226	0.0100	0.0237	1.023	1.003	1.043
****FEMALE****	Whole	NO_MAX	4	-0.0546	0.0200	0.0064	0.947	0.910	0.985
****FEMALE****	NSTEMI	CO_AVE6	1	0.0311	0.0143	0.0301	1.032	1.003	1.061
****FEMALE****	HTN	NO2_AVE	1	0.1007	0.0401	0.0122	1.106	1.022	1.196
****FEMALE****	HTN	NO2_AVE6	1	0.1120	0.0398	0.0049	1.118	1.035	1.209
****FEMALE****	Dysrhy	NO2_AVE6	5	-0.1590	0.0519	0.0022	0.853	0.770	0.944
****FEMALE****	PIHD	O3_MAX	0	-0.0751	0.0336	0.0254	0.928	0.869	0.991
****AGECAT1****	NSTEMI	O3_AVE12	5	-0.0763	0.0282	0.0069	0.927	0.877	0.979
****AGECAT1****	Diabetes	NO_AVE6	5	-0.0459	0.0216	0.0339	0.955	0.916	0.997
****AGECAT2****	Whole	NO2_AVE	1	0.0678	0.0255	0.0079	1.070	1.018	1.125
****AGECAT2****	Whole	NO2_AVE12	1	0.0724	0.0260	0.0053	1.075	1.022	1.131
****AGECAT2****	Whole	NO2_AVE6	1	0.0871	0.0254	0.0006	1.091	1.038	1.147
****AGECAT2****	NSTEMI	CO_AVE12	1	-0.0607	0.0224	0.0066	0.941	0.901	0.983
****AGECAT2****	NSTEMI	CO_AVE6	1	-0.0542	0.0236	0.0217	0.947	0.904	0.992
****AGECAT2****	NSTEMI	NO_AVE	1	0.0340	0.0165	0.0398	1.035	1.002	1.069
****AGECAT2****	NSTEMI	NO_AVE12	1	0.0326	0.0155	0.0350	1.033	1.002	1.065
****AGECAT2****	NSTEMI	NO_AVE6	1	0.0301	0.0145	0.0375	1.031	1.002	1.060
****AGECAT2****	NSTEMI	NO2_AVE	1	0.1028	0.0343	0.0027	1.108	1.036	1.185
****AGECAT2****	NSTEMI	NO2_AVE12	1	0.1118	0.0338	0.0009	1.118	1.047	1.195
****AGECAT2****	NSTEMI	NO2_AVE6	1	0.1257	0.0342	0.0002	1.134	1.060	1.212
****AGECAT2****	NSTEMI	NO2_MIN	3	0.0507	0.0191	0.0079	1.052	1.013	1.092
****AGECAT2****	Diabetes	NO_MAX	2	0.0520	0.0166	0.0018	1.053	1.020	1.088
****AGECAT2****	HTN	NO2_AVE	1	0.1117	0.0329	0.0007	1.118	1.048	1.193
****AGECAT2****	HTN	NO2_AVE12	1	0.0946	0.0302	0.0017	1.099	1.036	1.166
****AGECAT2****	HTN	NO2_AVE6	1	0.1152	0.0326	0.0004	1.122	1.053	1.196
****AGECAT2****	HTN	NO2_MAX	1	0.0427	0.0196	0.0295	1.044	1.004	1.084

Note: AGECAT1 = age <65; AGECAT2 = age ≥65. NSTEMI = Non-ST Segment Elevation Myocardial Infarction; Dysrhy = Dysrhythmia; HTN = Hypertension; AVE = 24-hour average; Ave6 = 6-hour average, AVE12 = 12-hour average, MAX = maximum 1-hour; MIN = minimum 1-hour. Data were calculated for an inter-quartile range increase of CO_AVE (0.35 mg/m^3^), CO_AVE12 (0.35 mg/m^3^), CO_AVE6 (0.40 mg/m^3^), CO_MIN (0.12 mg/m^3^), CO_MAX (0.81 mg/m^3^), NO_AVE (23.8 μg/m^3^), NO_AVE12 (25.2 μg/m^3^), NO_AVE6 (30.8 μg/m^3^), NO_MIN (2.5 μg/m^3^), NO_MAX (85 μg/m^3^), NO2_AVE (28.2 μg/m^3^), NO2_AVE12 (30.1 μg/m^3^), NO2_AVE6 (34.2 μg/m^3^), NO2_MIN (16.9 μg/m^3^), NO2_MAX (39.5 μg/m^3^), O3_AVE (30 μg/m^3^), O3_AVE12 (37.7 μg/m^3^), O3_AVE6 (37.7 μg/m^3^), O3_MIN (14 μg/m^3^), O3_MAX (36 μg/m^3^), PM25_AVE (7.7 μg/m^3^), PM25_AVE12 (8.5 μg/m^3^), PM25_AVE6 (8.8 μg/m^3^), PM25_MIN (3.1 μg/m^3^), PM25_MAX (17 μg/m^3^).

**Table 3:** Step 3 Bootstrap model averaging results.

				Median of 1,000 bootstrap estimates^a^						
Cohort	Subgroup	Variable	Lag (day)	Coefficient	StdErr	p-value	OR	Lower CL	Upper CL	Frequency^b^
**MAIN**	Whole	NO2_Ave12	1	0.0499	0.0174	0.0043	1.051	1.016	1.087	769
**MAIN**	Whole	NO2_Ave6	1	0.0487	0.0178	0.0075	1.050	1.013	1.087	710
**MAIN**	Diabetes	O3_MIN	1	0.0297	0.0166	0.0797	1.030	1.000	1.065	435
**MAIN**	HTN	NO2_Ave	1	0.0782	0.0252	0.0016	1.081	1.029	1.134	889
**MAIN**	HTN	NO2_AVE12	1	0.0771	0.0250	0.0017	1.080	1.029	1.133	856
**MAIN**	HTN	NO2_Ave6	1	0.0747	0.0251	0.0030	1.078	1.026	1.132	836
**MAIN**	Dysrhy	PM25_Ave	0	-0.0447	0.0186	0.0161	0.956	0.922	0.992	681
**MAIN**	Dysrhy	PM25_Ave12	0	-0.0423	0.0182	0.0195	0.959	0.925	0.993	672
**MAIN**	Dysrhy	PM25_Max	0	-0.0348	0.0164	0.0336	0.966	0.935	0.997	568
**MALE**	Dysrhy	PM25_Ave	0	-0.0577	0.0232	0.0133	0.944	0.902	0.988	716
**MALE**	Dysrhy	PM25_Ave12	0	-0.0606	0.0233	0.0081	0.941	0.899	0.984	756
**FEMALE**	Whole	CO_Ave6	1	0.0000	0.0000	1.0000	1.000	1.000	1.000	277
**FEMALE**	Whole	NO_Max	4	-0.0614	0.0204	0.0023	0.940	0.904	0.978	789
**FEMALE**	NSTEMI	CO_AVE6	1	0.0000	0.0000	1.0000	1.000	1.000	1.000	139
**FEMALE**	HTN	NO2_AVE	1	0.0963	0.0395	0.0180	1.101	1.017	1.189	619
**FEMALE**	HTN	NO2_Ave6	1	0.1099	0.0407	0.0088	1.116	1.028	1.210	688
**FEMALE**	Dysrhy	NO2_Ave6	5	-0.0785	0.0505	0.2369	0.925	0.819	1.000	305
**FEMALE**	PIHD	O3_MAX	0	0.0000	0.0000	1.0000	1.000	1.000	1.000	87
**AGECAT1**	NSTEMI	O3_Ave12	5	-0.0578	0.0285	0.0605	0.944	0.889	1.000	484
**AGECAT1**	Diabetes	NO_Ave6	5	-0.0461	0.0221	0.0513	0.955	0.911	1.000	497
**AGECAT2**	Whole	NO2_Ave	1	0.0717	0.0253	0.0035	1.074	1.024	1.127	823
**AGECAT2**	Whole	NO2_Ave12	1	0.0724	0.0250	0.0035	1.075	1.024	1.130	801
**AGECAT2**	Whole	NO2_Ave6	1	0.0885	0.0257	0.0006	1.092	1.039	1.148	935
**AGECAT2**	NSTEMI	CO_Ave12	1	-0.0596	0.0217	0.0056	0.942	0.903	0.983	855
**AGECAT2**	NSTEMI	CO_Ave6	1	-0.0527	0.0227	0.0178	0.949	0.907	0.991	663
**AGECAT2**	NSTEMI	NO_Ave	1	0.0338	0.0172	0.0549	1.034	1.000	1.071	489
**AGECAT2**	NSTEMI	NO_Ave12	1	0.0314	0.0161	0.0566	1.032	1.000	1.066	478
**AGECAT2**	NSTEMI	NO_Ave6	1	0.0327	0.0148	0.0300	1.033	1.003	1.065	585
**AGECAT2**	NSTEMI	NO2_Ave	1	0.1084	0.0351	0.0017	1.114	1.041	1.194	904
**AGECAT2**	NSTEMI	NO2_Ave12	1	0.1107	0.0349	0.0016	1.117	1.042	1.196	867
**AGECAT2**	NSTEMI	NO2_Ave6	1	0.1172	0.0350	0.0008	1.124	1.050	1.205	920
**AGECAT2**	NSTEMI	NO2_Min	3	0.0529	0.0238	0.0250	1.054	1.007	1.105	615
**AGECAT2**	Diabetes	NO_Max	2	0.0653	0.0247	0.0126	1.067	1.014	1.124	646
**AGECAT2**	HTN	NO2_Ave	1	0.1079	0.0317	0.0005	1.114	1.047	1.185	921
**AGECAT2**	HTN	NO2_AVE12	1	0.1049	0.0324	0.0008	1.111	1.044	1.184	884
**AGECAT2**	HTN	NO2_Ave6	1	0.1082	0.0320	0.0005	1.114	1.049	1.186	902
**AGECAT2**	HTN	NO2_Max	1	0.0691	0.0261	0.0098	1.072	1.017	1.128	691

Note: AGECAT1 = age <65; AGECAT2 = age ≥65. NSTEMI = Non-ST Segment Elevation Myocardial Infarction; HTN = Hypertension; Dysrhy = Dysrhythmia; AVE = 24-hour average; AVE6 = 6-hour average, AVE12 = 12-hour average, MAX = maximum 1-hour; MIN = minimum 1-hour. Data were calculated for an inter-quartile range increase of CO_AVE (0.35 mg/m^3^), CO_AVE12 (0.35 mg/m^3^), CO_AVE6 (0.40 mg/m^3^), CO_MIN (0.12 mg/m^3^), CO_MAX (0.81 mg/m^3^), NO_AVE (23.8 μg/m^3^), NO_AVE12 (25.2 μg/m^3^), NO_AVE6 (30.8 μg/m^3^), NO_MIN (2.5 μg/m^3^), NO_MAX (85 μg/m^3^), NO2_AVE (28.2 μg/m^3^), NO2_AVE12 (30.1 μg/m^3^), NO2_AVE6 (34.2 μg/m^3^), NO2_MIN (16.9 μg/m^3^), NO2_MAX (39.5 μg/m^3^), O3_AVE (30 μg/m^3^), O3_AVE12 (37.7 μg/m^3^), O3_AVE6 (37.7 μg/m^3^), O3_MIN (14 μg/m^3^), O3_MAX (36 μg/m^3^), PM25_AVE (7.7 μg/m^3^), PM25_AVE12 (8.5 μg/m^3^), PM25_AVE6 (8.8 μg/m^3^), PM25_MIN (3.1 μg/m^3^), PM25_MAX (17 μg/m^3^).

^a^ Median value from 1,000 model replications.

^b^ Number of times that a variable was significant (p-value ≤0.05) from 1,000 model replications.

**Fig 2:**
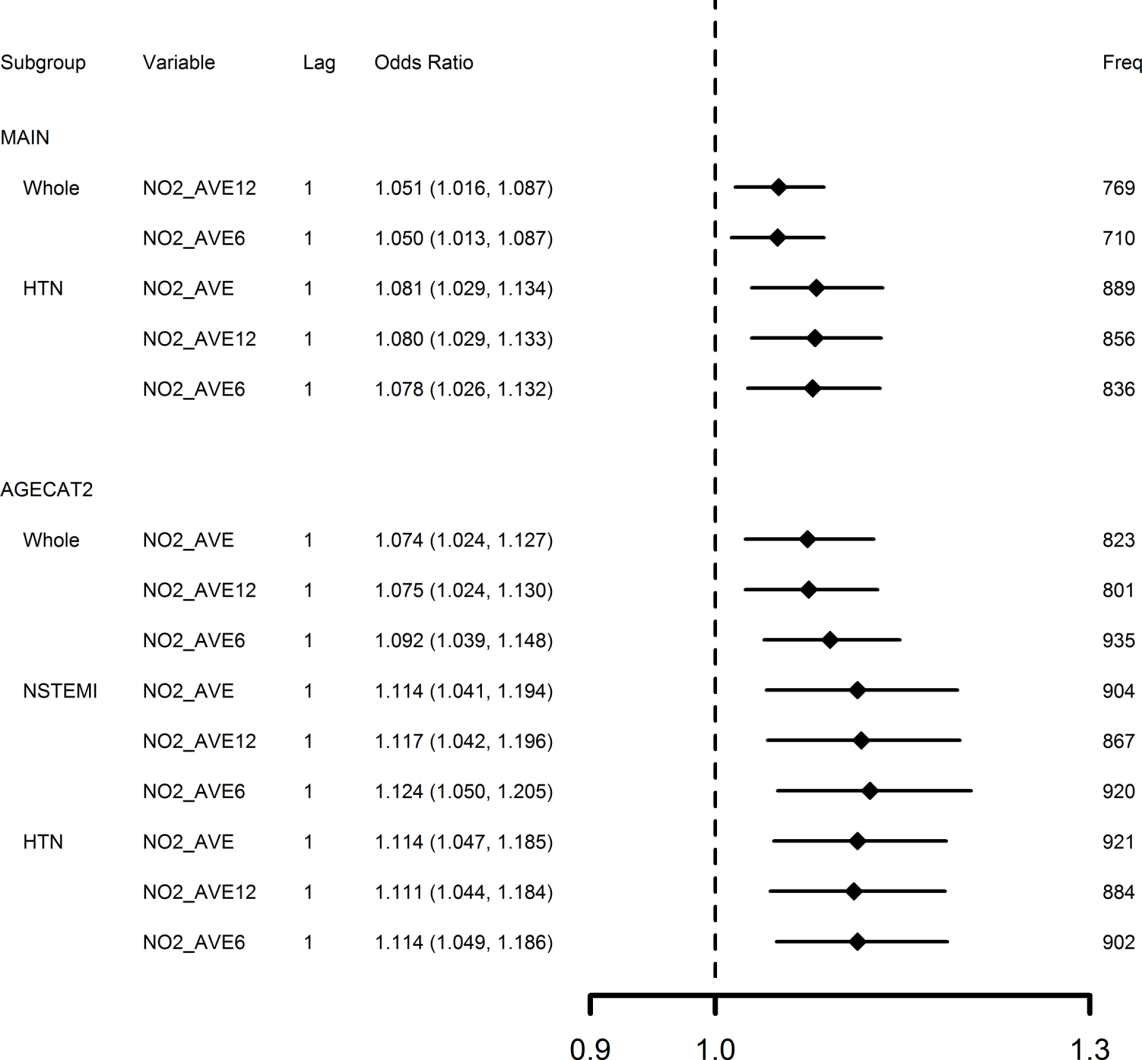
Adverse associations between air pollutants and AMI hospitalizations with frequency over 700 of 1,000 bootstrap replications. AGECAT2 = age ≥65; NSTEMI = Non-ST Segment Elevation Myocardial Infarction; HTN = Hypertension. OR estimates calculated by bootstrap model averaging of 1,000 replications for an inter-quintile range increase of NO2_AVE (28.2 μg/m^3^), NO2_AVE12 (30.1 μg/m^3^), NO2_AVE6 (34.2 μg/m^3^). Freq represents frequency that a variable had p-value ≤0.05 from 1,000 model replications.

We further identified those variables with positive associations and a bootstrap frequency over 700 as the most robust findings of the study (summarized in Fig 2). From these positive robust associations we observed: (1) only 1-day lag NO_2_ concentrations (6-, 12- or 24-hour average), but not those of CO, NO, O_3_ or PM_2.5_, were associated with an elevated risk of AMI hospitalization; (2) evidence was suggested for an effect of elevated risk of hospitalization for NSTEMI, but not for STEMI from increased NO_2_ concentrations; and (3) subgroups susceptible to increased NO_2_ concentrations included elders (age ≥65) and elders with hypertension. The most robust association (bootstrap frequency 935) was for an IQR increase of 34.2 μg/m^3^ in the 6-hour average NO_2_ concentrations 1-day before that elevated risk of AMI hospitalization 9.2% (95% CI 3.9% to 14.8%).

**Fig 3:**
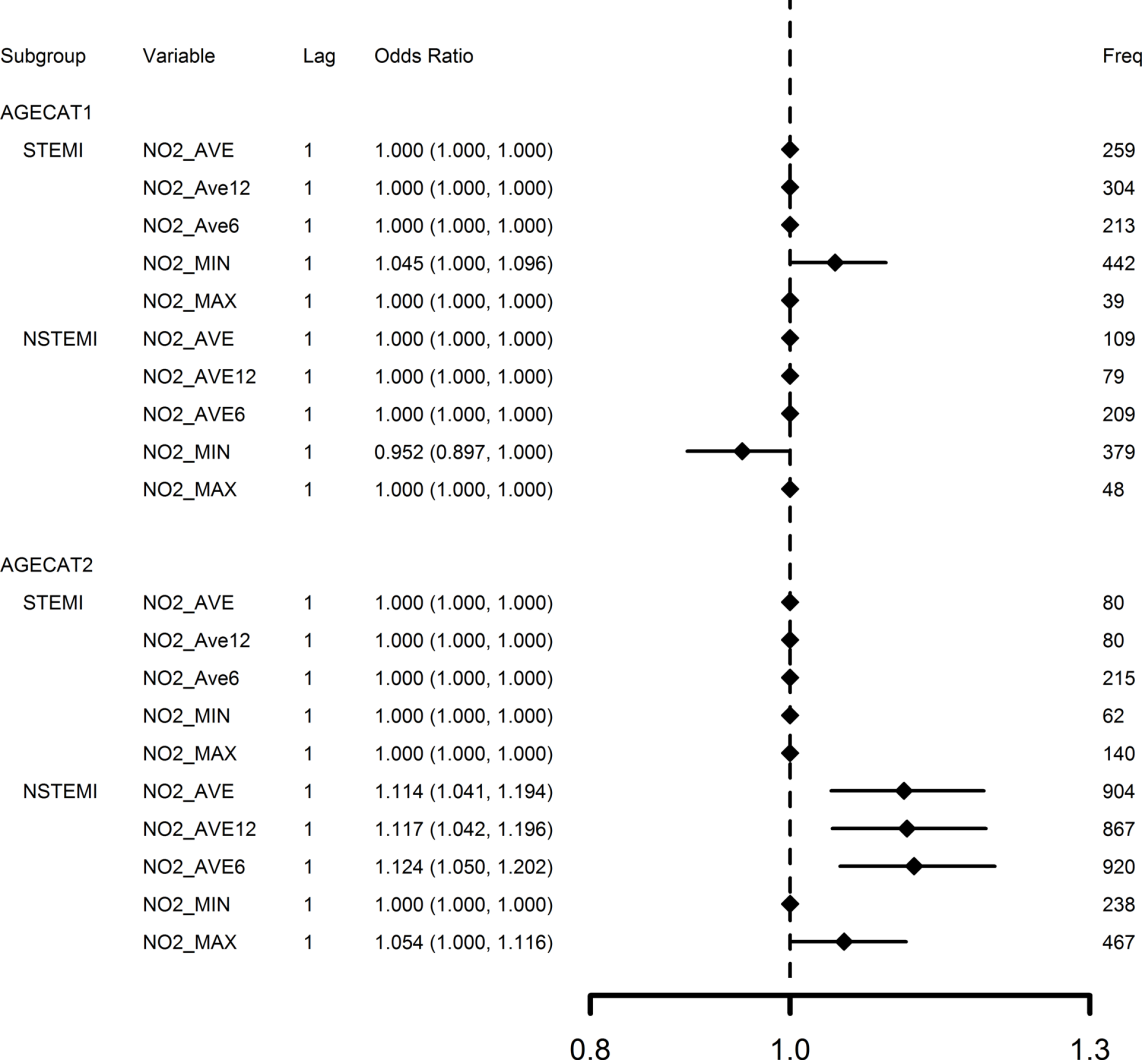
Comparison of 1-day lag NO_2_ concentration effects in subgroups defined by age and type of AMI. AGECAT1 = age <65; AGECAT2 = age ≥65; STEMI = ST Segment Elevation Myocardial Infarction; NSTEMI = Non-ST Segment Elevation Myocardial Infarction. OR estimates calculated by bootstrap model averaging of 1000 replications for an inter-quintile range increase of NO2_AVE (28.2 μg/m^3^), NO2_AVE12 (30.1 μg/m^3^), NO2_AVE6 (34.2 μg/m^3^), NO2_MIN (16.9 μg/m^3^), NO2_MAX (39.5 μg/m^3^). Freq represents frequency that a variable had p-value ≤0.05 from 1,000 model replications.

**Fig 4:**
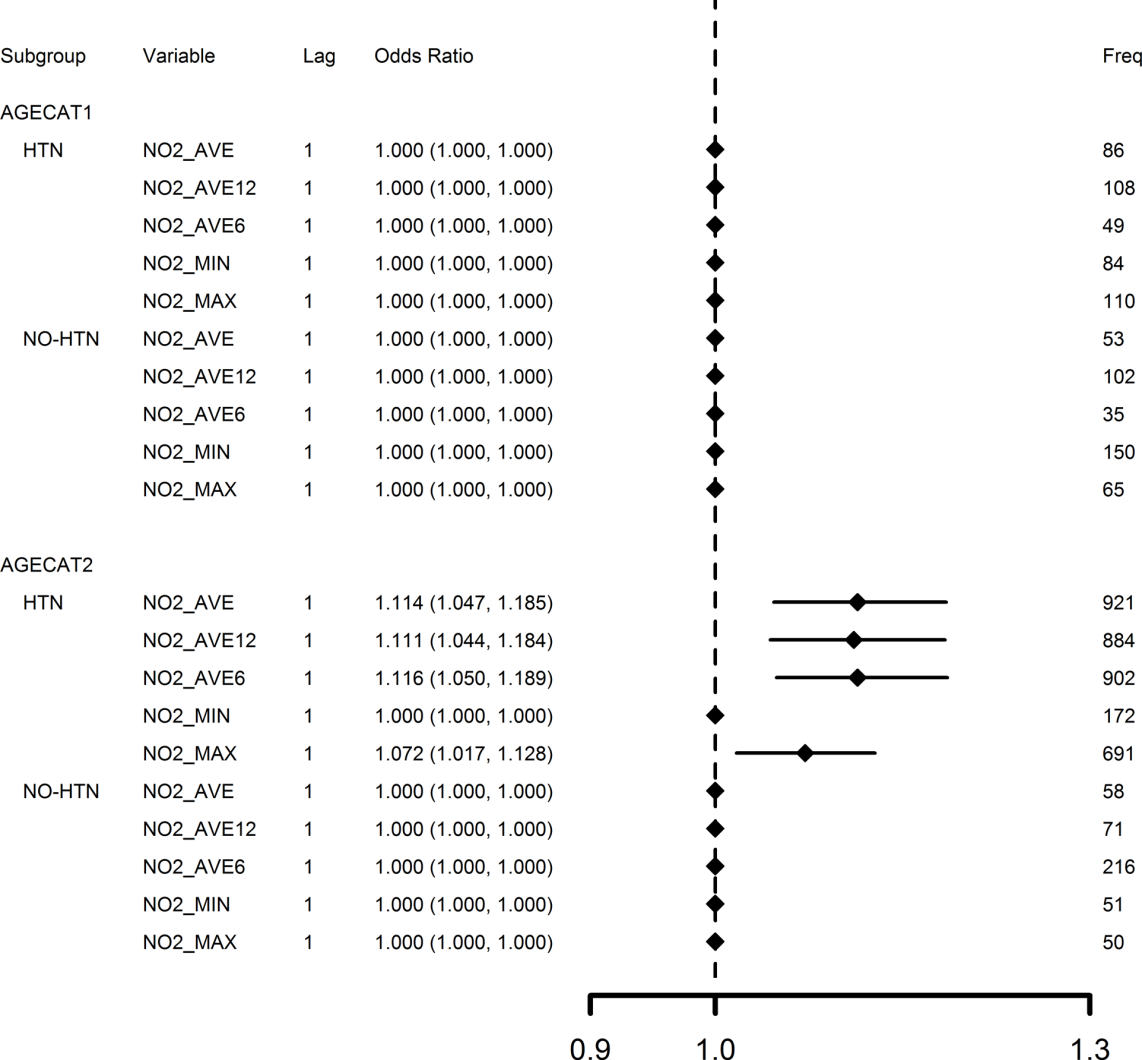
Comparison of 1-day lag NO_2_ concentration effects in subgroups defined by age and hypertension condition. AGECAT1 = age <65; AGECAT2 = age ≥65; HTN = Hypertension. NO-HTN = No Hypertension. OR estimates calculated by bootstrap model averaging of 1,000 replications for an inter-quintile range increase of NO2_AVE (28.2 μg/m^3^), NO2_AVE12 (30.1 μg/m^3^), NO2_AVE6 (34.2 μg/m^3^), NO2_MIN (16.9 μg/m^3^), NO2_MAX (39.5 μg/m^3^). Freq represents frequency that a variable had p-value ≤0.05 from 1,000 model replications.

Effects of the five measures of NO_2_ (with 1-day lag) in the four subgroups defined by age categories (agecat1 and 2) and AMI type (STEMI and NSTEMI) are compared in Fig 3 for OR results estimated from bootstrapping (Step 3). NO_2_ concentration increases were mainly associated with NSTEMI, instead of STEMI; and elders (age≥65) were suggested be more susceptible to increased NO_2_ concentration. Effects of the five measures of NO_2_ (with 1-day lag) in the four subgroups defined by age and hypertension conditions—with (HTN) or without (NO-HTN)–are also compared in Fig 4 for OR results estimated from bootstrapping. Fig 4 further supports that NO_2_ concentration increases were associated with hypertension for elders (age≥65).

The largest air pollutant dataset used in our analysis was for NO_2_ (from 51 stations). Because of this, NO_2_ can be considered as a proxy air pollutant to assess spatial variation in exposure to the ambient air pollutant mixture. Our most robust air pollutant associations with AMI were for NO_2_ and this may be because NO_2_ is the most representative air pollutant for exposure assessment. To better understand whether our findings were sensitive to the size of the cohort, we undertook further analysis using two smaller distance categories to identify patients hospitalized that were living within 5 km and within 10 km of the closest effective air monitoring station and then compared results to our original analysis that used patients hospitalized that were living within 15 km of the closest effective air monitoring station. Specifically, using the same data we linked patients living within 5 km (and 10 km) of the closest effective air monitoring station to air pollution data and then performed our three-step procedure for each distance category cohort.

The 5-km distance category had the smallest cohort size (13,071 hospitalization records), while the 10-km distance category included another 9,127 records (total 22,198) (see Table C in S1 File) compared to the original of 25,894 hospitalization records for all patients within a 15-km distance of the closest effective air monitoring station (Table 1). Bootstrap model averaging results for cohorts in the 5-km and 10-km distance categories (see Table D in S1 File) indicated that sample size influenced our findings. The smallest cohort within 5 km (13,071 hospitalization records) suggested positive associations with O_3_ and an elevated risk of AMI hospitalizations for bootstrap frequencies over 700; whereas these associations were lost for the larger sample sizes within 10 km and 15 km (22,198 and 25,894 hospitalization records, respectively). In addition, the only variable suggesting positive associations for bootstrap frequencies over 700 for the within 10-km samples (Table D in S1 File) was NO_2_ –similar to what was found in the original analysis (Table 3). Whereas there was a large increment in sample size going from patients living within 5 km to patients living within 10 km of an effective air monitoring station (70% increase), there was only a small increment in sample size going from patients living within 10 km to our original cohort (<17% increase). Thus a similar finding of positive associations with NO_2_ for bootstrap frequencies over 700 for patients living within 10 km compared to our original analysis was expected.

## Discussion

The most robust results in our study, after controlling for adjustment uncertainty and model uncertainty, suggest that only NO_2_ –but not the other air pollutant investigated including CO, NO, O_3_ or PM_2.5_ –was associated with elevated risk of NSTEMI hospitalization. These findings are consistent with a recent observational study of a very large sample of 452,343 MI cases [1] and several other studies [11, 17–19, 22]. The suggested finding of NSTEMI, not STEMI, associated with increasing NO_2_ concentrations is contrary with findings of others [3] in which it was reported that ambient fine particulate air matter (PM_2.5_) triggers STEMI, but not NSTEMI.

Our findings also suggest that elders (age ≥65) with hypertension are more susceptible. Despite numerous studies indicating elders to be more susceptible to increased air pollution [3,6,11,13,19,20,22,29], only one study [3] indicated people with hypertension could be more susceptible to increased particle concentrations. To the best of our knowledge, we have not seen finding like ours in literature about elders with hypertension being at increased risk to NO_2_ pollution. Because studies of air pollution effects on MI in people with hypertension are unusual, it is hard to compare our results with previous research. It is also unclear what mechanisms of action may be behind this suggested effect. Finally, our study did not see evidence that people age <65 years or those with pre-existing diabetes/dysrhythmia/prehistory of heart disease were susceptible to increased pollution.

We caution readers about preliminary findings suggesting associations of NO_2_ with elevated risk of NSTEMI hospitalization and AMI hospitalizations of elders (age ≥65) with hypertension. This was only an exploratory study and the emphasis was on application of a methodology to address adjustment and model uncertainty. There is clearly a need to replicate these preliminary findings using other approaches and/or different datasets in order to corroborate the suggested associations with NO_2_.

The general lack of a robust air pollution effect on risk of AMI (especially STEMI) in our analysis is not unexpected. Although a recent systematic review reported that most air pollutants were associated with increased short-term risk for MI [31], a previous review [49] indicated that less than half of the identified studies found clear evidence of raised MI risk from air pollutant exposure. Also, the fully adjusted associations in our analysis may be very different from those estimated with limited adjustment. For example, eight studies reported in Table A in S1 File associated increasing PM_2.5_ concentrations with increased risk for MI [3–5,7,15,18,19,23]; whereas in our analysis—which included full adjustment and model averaging—the effects of PM_2.5_ were negative (Table 3). We fully agree with recommendations of others [33,37] about the importance of focusing on estimating health effects that are properly adjusted for all confounding factors. We also highlight the need to consider controlling for model uncertainty as we found that 9 of 37 fully-adjusted associations lost statistical significance after bootstrapping in our analysis.

Our exploratory study had a number of strengths that address important limitations in the investigation of subtle air pollution-acute health effect associations. We used large air pollutant and meteorological datasets consisting of multiple locations and 11 years of AMI hospitalization records collected throughout Alberta. We only considered patients living 15 km or less to the closest effective air pollution monitoring station in Alberta. Most importantly, confounding from lack of adjustment and model uncertainty were examined by using fully adjusted models and bootstrap model averaging, and only the most robust findings were considered important. This was an ecological study with the exposure variables (air pollutants and meteorological variables) measured at central locations, and thus they do not represent actual exposures for AMI patients. Although each of the multivariate models was adjusted with possible air pollutant and meteorological confounders, because of data limitations we did not consider other potentially important time-varying factors such as SO_2_, special events (e.g., alcohol consumption, physical activity) or special drug usage just prior to the onset of AMI. Because SO_2_ may be an important criteria air pollutant in urban areas, further study of potential associations between air pollutants, including SO_2_, and AMI hospital admission is suggested.

## Conclusions

Estimating health effects that are properly adjusted for all possible confounding factors and accounting for model uncertainty are important for making interpretations of air pollution–health effect associations. The most robust statistical associations in our analysis were suggested for increasing 6-, 12- or 24-hour average concentrations of NO_2_ with 1-day lag and hospitalization for NSTEMI in Alberta. In addition, elderly people with hypertension were suggested to be at increased risk. As this was only an exploratory study there is a need to replicate these findings with other methodologies and datasets.

## Supporting Information

S1 File (DOCX)
