# Supplementary material for: Air Pollution and Acute Myocardial Infarction Hospital Admission in Alberta, Canada: A Three-Step Procedure Case-Crossover Study
Source: PLoS One. 2015 Jul 13;10(7):e0132769. doi: 10.1371/journal.pone.0132769 (PMC4500548; doi:10.1371/journal.pone.0132769)
Supplement: S1 File — (DOCX) [file pone.0132769.s001.docx]

**Supporting Information**

Air Pollution and Acute Myocardial Infarction Hospital Admission in Alberta, Canada: A Three-Step Procedure Case-Crossover Study

Xiaoming Wang^1,#^, Warren Kindzierski^2^* and Padma Kaul^3^

^1^ School of Public Health, University of Alberta, Edmonton, Alberta, Canada

^2^ School of Public Health, University of Alberta, Edmonton, Alberta, Canada

^3^ Canadian Vigour Centre, Department of Medicine, University of Alberta, Edmonton, Alberta, Canada

^#^ Current Address: Alberta Health Services, Research Innovation & Analytics, 7235- 2nd Floor, West Wing, 11402 University Avenue, Edmonton, Alberta, Canada

* Corresponding author

E-mail: warrenk@ualberta.ca

11 pages, 1 figure, 4 tables

**Supporting Information Captions**

**S1 File**. S1 File contains further details on: the locations of NAPS stations and NCDC stations in Alberta, Canada with records available in the study period (April 1, 1999-March 31, 2009) (**Figure A**); reviewed case-crossover studies in literature for association between particulate matter (PM) and myocardial infarction (MI) (**Table A**); nonparametric Wilcoxon test results from univariate analysis (Step 1) with p-value ≤0.1 (**Table B**); number of hospitalizations for acute myocardial infarction in Alberta subgroups living within a) 5 km, and b) 10 km of the closest effective air monitoring station (1999–2009) (**Table C**); and step 3 Bootstrap model averaging results for cohort living within a) 5 km, and b) 10 km of the closest effective air monitoring station (1999–2009) (**Table D**).

**Figure A. Locations of NAPS stations (left panel) and NCDC stations (right panel) in Alberta, Canada with records available in the study period (April 1, 1999-March 31, 2009).**

**Table A. Review of case-crossover studies in literature for association between particulate matter (PM) and myocardial infarction (MI).**

**Table B. Nonparametric Wilcoxon test results from univariate analysis (Step 1) with p‑value ≤0.1.**

**Table C. Number of hospitalizations for acute myocardial infarction in Alberta subgroups living within a) 5 km, and b) 10 km of the closest effective air monitoring station (1999–2009).**

**Table D. Step 3 Bootstrap model averaging results for cohort living within a) 5 km, and b) 10 km of the closest effective air monitoring station (1999–2009).**

**Figure A. Locations of NAPS stations (left panel) and NCDC stations (right panel) in Alberta, Canada with records available in the study period (April 1, 1999-March 31, 2009).**

**Table A. Review of case-crossover studies in literature for association between particulate matter (PM) and myocardial infarction (MI).**

| Study | Location | Subjects | Exposure | Design | Findings | Adjustment |
| --- | --- | --- | --- | --- | --- | --- |
| Milojevic et al. 2014 [1] | England and Wales | 452,343 cases of MI | PM10, PM2.5 | time-stratified | Not found | Adjusted for Temp, second pollutant if significant. |
| Bard et al. 2014 [2] | Strasbourg, France | 2,134 cases of MI | PM10 | time-stratified | Not found | Adjusted for Temp, AP and RH |
| Gardner et al. 2014 [3] | USA | 338 acute STEMI and 339 acute NSTEMI | PM(<2.5 um, 10-100 nm, 100-500 nm) | time-stratified | PM2.5 (1hour before onset) was associated with STEMI, but not NSTEMI | Adjusted for Temp and RH |
| Hodas et al. 2013 [4] | New Jersey, USA | 1,561 HA for Transmural MI (age ≥18) | PM2.5 | time-stratified | Refined ambient PM2.5 (24-hour average before onset) was associated with transmural MI | Adjusted for Tapp and residential air exchange rate |
| Rich et al. 2013 [5] | New Jersey, USA | 1,562 HA for transmural MI (age ≥18) | PM2.5 | time-stratified | PM2.5 (24-hour average before onset) was associated with transmural MI | Adjusted for Tapp |
| Kioumourtzoglou et al. 2013 [6] | 3 cities, USA | Emergency HA | OC | modified bidirectional | Not found | Adjusted for season, DOW, RH and Temp |
| Rosenthal et al. 2013 [7] | Helsinki, Finland | 629 cases of MI | PM10, PM2.5 | time-stratified | PM2.5(lag 0-hour,0-day) was associated with MI | Uni- or bi-pollutant model adjusted for RH and Temp |
| Tsai et al. 2012 [8] | Taipei, Taiwan | 27,563 HA for acute MI | PM10 | time-stratified | Not found | adjust for RH and Temp |
| Turin et al. 2012 [9] | Shiga, Japan | 429 first-time acute MI | SPM | bidirectional | Not found | Uni- or bi-pollutant model adjusted for RH and Temp |
| Bhaskaran et al. 2011 [10] | England & Wales | 79,288 HA for MI | PM10 | time-stratified | PM10(1-6hours average before onset) was associated with acute MI | Adjusted for Temp, RH, daily levels of influenza and respiratory syncytial virus |
| Nuvolone et al. 2011 [11] | Florence, Italy | 11,450 HA for acute MI | PM10 | time-stratified | PM10(lag 2-day) was associated with acute MI | Uni- or bi-pollutant model adjusted for holiday, influenza epidemics and population disease |
| von Klot et al. 2011 [12] | Augsburg, KORA | 960 AMI patients (age 25-74) | Soot, PM2.5 | unidirectional | Soot (but not PM2.5) was associated with acute MI | Adjusted for Temp and DOW |
| Cadum et al. 2009 [13] | 10 Italian cities | HA for acute MI | PM10 | time-stratified | PM10 was associated with acute MI | Unknown (article in Italian) |
| Berglind et al. 2010 [14] | Stockholm, Sweden | 660 first-time MI | PM10 | time-stratified | Not found | Adjusted for RH and Temp |
| Rich et al. 2010 [15] | New Jersey, USA | 5,864 HA for 1st-time AMI | PM2.5 | time-stratified | PM2.5(24-hour average before onset) was associated with transmural MI | Adjusted for Tapp |
| Hsieh et al. 2010 [16] | Taipei, Taiwan | 23,420 HA for MI | PM10 | time-stratified | PM10 (3-day average before onset) was associated with MI | Uni- or bi-pollutant model adjusted for RH and Temp |
| Cheng et al 2009 [17] | Kaohsiung, Taiwan | 9,349 HA for MI | PM10 | time-stratified | PM10 (3-day average before onset) in cold weather was associated with MI | Adjusted for RH and Temp |
| Zanobetti and Schwartz 2006 [18] | Boston, USA | 15,578 HA for acute MI | PM2.5, BC | time-stratified | PM2.5 (lag p-day) was associated with acute MI | Matched Tapp in case-crossover design |
| Barnett et al. 2006 [19] | 5 cities in Australian and New Zealand | HA for CVD (age ≥15) | PM2.5, PM10 | time-stratified | PM2.5(24hour average before onset) was associated with MI | Used a matched case-crossover approach |
| Study | **Location** | **Subjects** | **Exposure** | **Design** | **Findings** | **Adjustment** |
| Zanobetti and Schwartz 2005 [20] | 21 cities, USA | 302,453 HA for MI (age ≥65) | PM10 | time-stratified | PM10(lag 0day) was associated with MI | Adjusted for Tapp |
| Sullivan et al. 2005 [21] | Washington, USA | 5,793 cases of acute MI | PM2.5, PM10 | time-stratified | Not found | Adjusted for RH and Temp |
| D'Ippoliti et al. 2003 [22] | Rome, Italy | 6,531 HA for first-time AMI | TSP | time stratified | TSP(lag 0-to 2-day) was associated with acute MI | Adjusted for RH and Temp |
| Peters et al. 2001 [23] | Boston, USA | 772 Patients with MI | PM2.5, PM10, BC | unidirectional | PM2.5(lag 2-hour, 1-day) is associated with MI | Adjusted for Season, DOW, RH and Temp |

**Note: HA=hospital admission; BC=black carbon; TSP=total suspended particulate; Temp=temperature; Tapp= apparent temperature; RH=relative humidity; DOW=day of week; AP=atmospheric pressure; OC= organic carbon. Here we summarize only on the findings of associations between PM and MI for each study.**

**Table B. Nonparametric Wilcoxon test results from univariate analysis (Step 1) with p‑value ≤0.1.**

| **Cohort** | **Subgroup** | **Variable** | **Lag** | **p-value** |
| --- | --- | --- | --- | --- |
| MAIN | Whole | NO_AVE12 | 1 | 0.0983 |
| MAIN | Whole | NO2_AVE12 | 1 | 0.0477 |
| MAIN | Whole | NO2_AVE6 | 1 | 0.0978 |
| MAIN | STEMI | NO_AVE6 | 4 | 0.0522 |
| MAIN | STEMI | NO_MAX | 4 | 0.0994 |
| MAIN | STEMI | O3_MIN | 4 | 0.0947 |
| MAIN | STEMI | PM25_AVE | 2 | 0.0879 |
| MAIN | STEMI | PM25_AVE | 4 | 0.0552 |
| MAIN | STEMI | PM25_AVE6 | 4 | 0.0389 |
| MAIN | STEMI | PM25_MAX | 4 | 0.0233 |
| MAIN | NSTEMI | NO_AVE12 | 1 | 0.0366 |
| MAIN | NSTEMI | NO2_AVE12 | 1 | 0.0408 |
| MAIN | NSTEMI | PM25_AVE12 | 2 | 0.0771 |
| MAIN | NSTEMI | PM25_AVE12 | 4 | 0.0893 |
| MAIN | NSTEMI | PM25_AVE6 | 2 | 0.0397 |
| MAIN | NSTEMI | PM25_MIN | 4 | 0.0378 |
| MAIN | Diabetes | NO2_MAX | 2 | 0.0836 |
| MAIN | Diabetes | O3_MIN | 1 | 0.0380 |
| MAIN | Diabetes | PM25_AVE | 2 | 0.0383 |
| MAIN | Diabetes | PM25_AVE12 | 2 | 0.0312 |
| MAIN | Diabetes | PM25_AVE6 | 2 | 0.0143 |
| MAIN | Diabetes | PM25_MIN | 2 | 0.0222 |
| MAIN | Diabetes | PM25_MIN | 4 | 0.0357 |
| MAIN | Diabetes | PM25_MIN | 5 | 0.0694 |
| MAIN | Hypertension | NO_AVE12 | 1 | 0.0688 |
| MAIN | Hypertension | NO2_AVE | 1 | 0.0360 |
| MAIN | Hypertension | NO2_AVE12 | 1 | 0.0267 |
| MAIN | Hypertension | NO2_AVE6 | 1 | 0.0477 |
| MAIN | Hypertension | PM25_AVE12 | 2 | 0.0682 |
| MAIN | Hypertension | PM25_MIN | 2 | 0.0812 |
| MAIN | Dysrhythmia | O3_MIN | 2 | 0.0366 |
| MAIN | Dysrhythmia | PM25_AVE | 0 | 0.0269 |
| MAIN | Dysrhythmia | PM25_AVE | 2 | 0.0817 |
| MAIN | Dysrhythmia | PM25_AVE | 4 | 0.0253 |
| MAIN | Dysrhythmia | PM25_AVE12 | 0 | 0.0331 |
| MAIN | Dysrhythmia | PM25_AVE12 | 4 | 0.0839 |
| MAIN | Dysrhythmia | PM25_AVE6 | 4 | 0.0308 |
| MAIN | Dysrhythmia | PM25_MAX | 0 | 0.0998 |
| MAIN | Dysrhythmia | PM25_MAX | 2 | 0.0830 |
| MAIN | Dysrhythmia | PM25_MAX | 4 | 0.0053 |
| MALE | STEMI | PM25_AVE | 2 | 0.0881 |
| MALE | STEMI | PM25_AVE6 | 2 | 0.0965 |
| MALE | Diabetes | NO2_MAX | 2 | 0.0832 |
| MALE | Diabetes | O3_MIN | 1 | 0.0762 |
| MALE | Diabetes | PM25_AVE | 2 | 0.0506 |
| MALE | Diabetes | PM25_AVE12 | 2 | 0.0134 |
| MALE | Diabetes | PM25_AVE6 | 2 | 0.0218 |
| MALE | Diabetes | PM25_MIN | 2 | 0.0287 |
| MALE | Dysrhythmia | O3_MIN | 1 | 0.0890 |
| MALE | Dysrhythmia | PM25_AVE | 0 | 0.0463 |
| MALE | Dysrhythmia | PM25_AVE12 | 0 | 0.0384 |
| MALE | Dysrhythmia | PM25_MAX | 4 | 0.0795 |
| FEMALE | Whole | CO_AVE12 | 1 | 0.0636 |
| FEMALE | Whole | CO_AVE6 | 1 | 0.0359 |
| FEMALE | Whole | NO_AVE12 | 1 | 0.0590 |
| FEMALE | Whole | NO_AVE6 | 1 | 0.0688 |
| FEMALE | Whole | NO_MAX | 4 | 0.0821 |
| **Cohort** | **Subgroup** | **Variable** | **Lag** | **p-value** |
| FEMALE | Whole | NO2_AVE12 | 1 | 0.0746 |
| FEMALE | Whole | NO2_AVE6 | 1 | 0.0527 |
| FEMALE | Whole | O3_AVE | 1 | 0.0693 |
| FEMALE | Whole | O3_AVE12 | 1 | 0.0518 |
| FEMALE | Whole | O3_AVE6 | 1 | 0.0601 |
| FEMALE | Whole | O3_MAX | 1 | 0.0841 |
| FEMALE | Whole | PM25_MIN | 0 | 0.0632 |
| FEMALE | Whole | PM25_MIN | 4 | 0.0571 |
| FEMALE | STEMI | CO_MAX | 4 | 0.0264 |
| FEMALE | STEMI | NO_AVE | 4 | 0.0405 |
| FEMALE | STEMI | NO_AVE12 | 4 | 0.0715 |
| FEMALE | STEMI | NO_AVE6 | 4 | 0.0086 |
| FEMALE | STEMI | NO_MAX | 4 | 0.0197 |
| FEMALE | STEMI | NO2_AVE6 | 4 | 0.0991 |
| FEMALE | STEMI | O3_MIN | 4 | 0.0593 |
| FEMALE | STEMI | PM25_AVE | 4 | 0.0812 |
| FEMALE | STEMI | PM25_AVE12 | 4 | 0.0995 |
| FEMALE | STEMI | PM25_AVE6 | 4 | 0.0158 |
| FEMALE | STEMI | PM25_MAX | 3 | 0.0618 |
| FEMALE | STEMI | PM25_MAX | 4 | 0.0345 |
| FEMALE | NSTEMI | CO_AVE12 | 1 | 0.0646 |
| FEMALE | NSTEMI | CO_AVE6 | 1 | 0.0578 |
| FEMALE | NSTEMI | NO_AVE12 | 1 | 0.0651 |
| FEMALE | NSTEMI | NO2_AVE12 | 1 | 0.0866 |
| FEMALE | NSTEMI | O3_AVE12 | 1 | 0.0571 |
| FEMALE | NSTEMI | O3_AVE6 | 1 | 0.0890 |
| FEMALE | NSTEMI | PM25_MIN | 4 | 0.0108 |
| FEMALE | NSTEMI | PM25_MAX | 2 | 0.0835 |
| FEMALE | Diabetes | PM25_MIN | 4 | 0.0120 |
| FEMALE | Diabetes | PM25_MIN | 5 | 0.0062 |
| FEMALE | Hypertension | CO_AVE12 | 1 | 0.0514 |
| FEMALE | Hypertension | CO_AVE6 | 1 | 0.0468 |
| FEMALE | Hypertension | NO_AVE12 | 1 | 0.0953 |
| FEMALE | Hypertension | NO2_AVE | 1 | 0.0993 |
| FEMALE | Hypertension | NO2_AVE12 | 1 | 0.0765 |
| FEMALE | Hypertension | NO2_AVE6 | 1 | 0.0628 |
| FEMALE | Hypertension | PM25_MIN | 4 | 0.0078 |
| FEMALE | Dysrhythmia | CO_MAX | 4 | 0.0458 |
| FEMALE | Dysrhythmia | NO_MAX | 4 | 0.0854 |
| FEMALE | Dysrhythmia | NO2_AVE6 | 5 | 0.0671 |
| FEMALE | Dysrhythmia | O3_MIN | 2 | 0.0817 |
| FEMALE | Dysrhythmia | PM25_AVE | 4 | 0.0736 |
| FEMALE | Dysrhythmia | PM25_AVE6 | 4 | 0.0506 |
| FEMALE | Dysrhythmia | PM25_MAX | 4 | 0.0201 |
| FEMALE | PIHD | CO_AVE12 | 0 | 0.0957 |
| FEMALE | PIHD | NO2_MIN | 0 | 0.0546 |
| FEMALE | PIHD | O3_AVE | 1 | 0.0545 |
| FEMALE | PIHD | O3_AVE12 | 1 | 0.0252 |
| FEMALE | PIHD | O3_AVE6 | 1 | 0.0410 |
| FEMALE | PIHD | O3_MAX | 0 | 0.0755 |
| FEMALE | PIHD | O3_MAX | 1 | 0.0728 |
| AGECAT1 | STEMI | PM25_AVE | 2 | 0.0916 |
| AGECAT1 | STEMI | PM25_MAX | 2 | 0.0854 |
| AGECAT1 | NSTEMI | O3_AVE12 | 5 | 0.0767 |
| AGECAT1 | NSTEMI | O3_MIN | 4 | 0.0442 |
| AGECAT1 | NSTEMI | PM25_MIN | 4 | 0.0929 |
| AGECAT1 | Diabetes | CO_AVE6 | 4 | 0.0944 |
| AGECAT1 | Diabetes | CO_AVE6 | 5 | 0.0909 |
| AGECAT1 | Diabetes | CO_MAX | 5 | 0.0914 |
| AGECAT1 | Diabetes | NO_AVE6 | 5 | 0.0279 |
| AGECAT1 | Diabetes | NO2_MAX | 4 | 0.0522 |
| **Cohort** | **Subgroup** | **Variable** | **Lag** | **p-value** |
| AGECAT1 | Diabetes | PM25_MIN | 2 | 0.0400 |
| AGECAT1 | Hypertension | PM25_MIN | 2 | 0.0824 |
| AGECAT1 | Hypertension | PM25_MAX | 4 | 0.0579 |
| AGECAT1 | Dysrhythmia | PM25_MAX | 4 | 0.0625 |
| AGECAT1 | PIHD | PM25_AVE | 0 | 0.0740 |
| AGECAT1 | PIHD | PM25_AVE12 | 0 | 0.0586 |
| AGECAT1 | PIHD | PM25_AVE6 | 0 | 0.0803 |
| AGECAT1 | PIHD | PM25_MAX | 0 | 0.0147 |
| AGECAT2 | Whole | NO_AVE12 | 1 | 0.0452 |
| AGECAT2 | Whole | NO_AVE6 | 1 | 0.0513 |
| AGECAT2 | Whole | NO_MIN | 0 | 0.0580 |
| AGECAT2 | Whole | NO2_AVE | 1 | 0.0606 |
| AGECAT2 | Whole | NO2_AVE12 | 1 | 0.0383 |
| AGECAT2 | Whole | NO2_AVE6 | 1 | 0.0334 |
| AGECAT2 | Whole | PM25_MAX | 3 | 0.0789 |
| AGECAT2 | STEMI | NO_AVE | 4 | 0.0580 |
| AGECAT2 | STEMI | NO_AVE12 | 4 | 0.0602 |
| AGECAT2 | STEMI | NO_AVE6 | 4 | 0.0567 |
| AGECAT2 | STEMI | NO_MIN | 0 | 0.0440 |
| AGECAT2 | STEMI | O3_AVE | 4 | 0.0655 |
| AGECAT2 | STEMI | O3_AVE12 | 4 | 0.0803 |
| AGECAT2 | STEMI | O3_MIN | 4 | 0.0836 |
| AGECAT2 | STEMI | PM25_AVE | 4 | 0.0350 |
| AGECAT2 | STEMI | PM25_AVE12 | 4 | 0.0364 |
| AGECAT2 | STEMI | PM25_AVE6 | 4 | 0.0376 |
| AGECAT2 | STEMI | PM25_MAX | 4 | 0.0512 |
| AGECAT2 | NSTEMI | CO_AVE12 | 1 | 0.0828 |
| AGECAT2 | NSTEMI | CO_AVE6 | 1 | 0.0798 |
| AGECAT2 | NSTEMI | NO_AVE | 1 | 0.0576 |
| AGECAT2 | NSTEMI | NO_AVE12 | 1 | 0.0072 |
| AGECAT2 | NSTEMI | NO_AVE6 | 1 | 0.0134 |
| AGECAT2 | NSTEMI | NO_MAX | 1 | 0.0541 |
| AGECAT2 | NSTEMI | NO2_AVE | 1 | 0.0263 |
| AGECAT2 | NSTEMI | NO2_AVE12 | 1 | 0.0109 |
| AGECAT2 | NSTEMI | NO2_AVE6 | 1 | 0.0138 |
| AGECAT2 | NSTEMI | NO2_MIN | 3 | 0.0963 |
| AGECAT2 | NSTEMI | PM25_AVE6 | 1 | 0.0894 |
| AGECAT2 | Diabetes | CO_MAX | 2 | 0.0640 |
| AGECAT2 | Diabetes | NO_MAX | 2 | 0.0215 |
| AGECAT2 | Diabetes | NO2_AVE | 2 | 0.0962 |
| AGECAT2 | Diabetes | NO2_MAX | 2 | 0.0846 |
| AGECAT2 | Diabetes | PM25_AVE | 2 | 0.0142 |
| AGECAT2 | Diabetes | PM25_AVE | 4 | 0.0666 |
| AGECAT2 | Diabetes | PM25_AVE | 5 | 0.0828 |
| AGECAT2 | Diabetes | PM25_AVE12 | 2 | 0.0155 |
| AGECAT2 | Diabetes | PM25_AVE12 | 4 | 0.0224 |
| AGECAT2 | Diabetes | PM25_AVE12 | 5 | 0.0384 |
| AGECAT2 | Diabetes | PM25_AVE6 | 2 | 0.0052 |
| AGECAT2 | Diabetes | PM25_AVE6 | 4 | 0.0549 |
| AGECAT2 | Diabetes | PM25_MIN | 4 | 0.0417 |
| AGECAT2 | Diabetes | PM25_MIN | 5 | 0.0122 |
| AGECAT2 | Diabetes | PM25_MAX | 2 | 0.0282 |
| AGECAT2 | Hypertension | CO_AVE12 | 1 | 0.0783 |
| AGECAT2 | Hypertension | CO_AVE6 | 1 | 0.0798 |
| AGECAT2 | Hypertension | NO_AVE | 1 | 0.0762 |
| AGECAT2 | Hypertension | NO_AVE12 | 1 | 0.0245 |
| AGECAT2 | Hypertension | NO_AVE6 | 1 | 0.0245 |
| AGECAT2 | Hypertension | NO_MIN | 1 | 0.0507 |
| AGECAT2 | Hypertension | NO_MIN | 3 | 0.0764 |
| AGECAT2 | Hypertension | NO2_AVE | 1 | 0.0118 |
| AGECAT2 | Hypertension | NO2_AVE12 | 1 | 0.0094 |
| **Cohort** | **Subgroup** | **Variable** | **Lag** | **p-value** |
| AGECAT2 | Hypertension | NO2_AVE6 | 1 | 0.0090 |
| AGECAT2 | Hypertension | NO2_MAX | 1 | 0.0594 |
| AGECAT2 | Hypertension | PM25_AVE12 | 5 | 0.0541 |
| AGECAT2 | Hypertension | PM25_AVE6 | 5 | 0.0776 |
| AGECAT2 | Hypertension | PM25_MIN | 5 | 0.0534 |
| AGECAT2 | Dysrhythmia | O3_MIN | 2 | 0.0718 |
| AGECAT2 | Dysrhythmia | PM25_AVE | 0 | 0.0208 |
| AGECAT2 | Dysrhythmia | PM25_AVE | 4 | 0.0562 |
| AGECAT2 | Dysrhythmia | PM25_AVE12 | 0 | 0.0263 |
| AGECAT2 | Dysrhythmia | PM25_AVE6 | 4 | 0.0640 |
| AGECAT2 | Dysrhythmia | PM25_MAX | 0 | 0.0823 |
| AGECAT2 | Dysrhythmia | PM25_MAX | 4 | 0.0324 |
| AGECAT2 | PIHD | NO2_AVE12 | 1 | 0.0949 |

**Note**: STEMI=ST Segment Elevation Myocardial Infarction; NSTEMI=Non-ST Segment Elevation Myocardial Infarction; PIHD=prehistory of heart disease.

**Table C. Number of hospitalizations for acute myocardial infarction in Alberta subgroups living within a) 5 km, and b) 10 km of the closest effective air monitoring station (1999–2009).**

**a) living within 5 km of the closest effective air monitoring station**

| Cohort | Whole | STEMI | NSTEMI | Diabetes | HTN | Dysrhy | PIHD |
| --- | --- | --- | --- | --- | --- | --- | --- |
| MAIN | 13071 | 6349 | 6722 | 3157 | 7050 | 2394 | 3930 |
| MALE | 8590 | 4392 | 4198 | 2004 | 4288 | 1494 | 2531 |
| FEMALE | 4481 | 1957 | 2524 | 1153 | 2762 | 900 | 1399 |
| AGECAT1 | 5369 | 2981 | 2388 | 1088 | 2430 | 522 | 906 |
| AGECAT2 | 7702 | 3368 | 4334 | 2069 | 4620 | 1872 | 3024 |

**b) living within 10 km of the closest effective air monitoring station**

| Cohort | Whole | STEMI | NSTEMI | Diabetes | HTN | Dysrhy | PIHD |
| --- | --- | --- | --- | --- | --- | --- | --- |
| MAIN | 22198 | 10854 | 11344 | 5344 | 11900 | 3927 | 6402 |
| MALE | 14907 | 7629 | 7278 | 3438 | 7435 | 2523 | 4204 |
| FEMALE | 7291 | 3225 | 4066 | 1906 | 4465 | 1404 | 2198 |
| AGECAT1 | 9796 | 5424 | 4372 | 1948 | 4435 | 941 | 1632 |
| AGECAT2 | 12402 | 5434 | 6968 | 3396 | 7465 | 2986 | 4770 |

**Note: AGECAT1= age <65; AGECAT2=age ≥65; STEMI=ST Segment Elevation Myocardial Infarction; NSTEMI=Non-ST Segment Elevation Myocardial Infarction; HTN=Hypertension; Dysrhy=Dysrhythmia; PIHD=prehistory of heart disease.**

**Table D. Step 3 Bootstrap model averaging results for cohort living within a) 5 km, and b) 10 km of the closest effective air monitoring station (1999–2009).**

**a) living within 5 km of the closest effective air monitoring station**

| Cohort | Subgroup | Variable | Lag (day) | Median of 1,000 bootstrap estimates^a^ | | | | | | Frequency^b^ |
| --- | --- | --- | --- | --- | --- | --- | --- | --- | --- | --- |
|  |  |  |  | **Coefficient** | **StdErr** | **p-value** | **OR** | **Lower CL** | **Upper CL** |  |
| FEMALE | PIHD | O3_Max | 3 | 0.1336 | 0.0498 | 0.0066 | 1.143 | 1.039 | 1.261 | 732 |
| AGECAT1 | PIHD | O3_Min | 5 | 0.1161 | 0.0422 | 0.0059 | 1.123 | 1.035 | 1.219 | 799 |
| AGECAT2 | Diabetes | PM25_Ave12 | 5 | 0.0514 | 0.0256 | 0.0500 | 1.053 | 1.000 | 1.107 | 499 |

**Note: AGECAT1= age <65; AGECAT2=age ≥65. PIHD=prehistory of heart disease; AVE12=12-hour average, MAX=maximum 1-hour; MIN=minimum 1-hour. Data were calculated for an inter-quartile range increase of O3_MIN (7 µg/m^3^), O3_MAX (18 µg/m^3^), PM25_AVE12 (8.5 µg/m^3^).**

**b) living within 10 km of the closest effective air monitoring station**

| Cohort | Subgroup | Variable | Lag (day) | Median of 1,000 bootstrap estimates^a^ | | | | | | Frequency^b^ |
| --- | --- | --- | --- | --- | --- | --- | --- | --- | --- | --- |
|  |  |  |  | **Coefficient** | **StdErr** | **p-value** | **OR** | **Lower CL** | **Upper CL** |  |
| MAIN | NSTEMI | NO2_AVE12 | 1 | 0.0491 | 0.0234 | 0.0624 | 1.050 | 1.000 | 1.104 | 472 |
| MAIN | HTN | NO2_Ave12 | 1 | 0.0845 | 0.0272 | 0.0016 | 1.088 | 1.032 | 1.147 | 834 |
| MAIN | Dysrhy | O3_MIN | 1 | 0.0382 | 0.0205 | 0.0707 | 1.039 | 1.000 | 1.084 | 456 |
| MALE | Dysrhy | O3_Min | 1 | 0.0502 | 0.0248 | 0.0541 | 1.051 | 1.000 | 1.106 | 494 |
| FEMALE | NSTEMI | PM25_Ave12 | 4 | 0.0325 | 0.0187 | 0.0910 | 1.033 | 1.000 | 1.072 | 371 |
| FEMALE | HTN | PM25_MIN | 4 | 0.0231 | 0.0117 | 0.0484 | 1.023 | 1.000 | 1.047 | 506 |
| AGECAT1 | Whole | NO2_MIN | 0 | 0.0427 | 0.0199 | 0.0326 | 1.044 | 1.003 | 1.086 | 556 |
| AGECAT2 | NSTEMI | NO2_Ave | 1 | 0.1015 | 0.0376 | 0.0068 | 1.107 | 1.028 | 1.191 | 778 |
| AGECAT2 | NSTEMI | NO2_Ave12 | 1 | 0.1024 | 0.0373 | 0.0068 | 1.108 | 1.029 | 1.192 | 744 |
| AGECAT2 | NSTEMI | NO2_Ave6 | 1 | 0.1148 | 0.0377 | 0.0024 | 1.122 | 1.041 | 1.209 | 840 |
| AGECAT2 | HTN | NO2_Ave | 1 | 0.1145 | 0.0348 | 0.0007 | 1.121 | 1.050 | 1.199 | 900 |
| AGECAT2 | HTN | NO2_Ave12 | 1 | 0.1100 | 0.0346 | 0.0011 | 1.116 | 1.045 | 1.194 | 849 |
| AGECAT2 | HTN | NO2_Ave6 | 1 | 0.1177 | 0.0347 | 0.0006 | 1.125 | 1.051 | 1.204 | 905 |
| AGECAT2 | HTN | PM25_Min | 5 | 0.0188 | 0.0089 | 0.0360 | 1.019 | 1.001 | 1.037 | 555 |

**Note: AGECAT1= age <65; AGECAT2=age ≥65. NSTEMI=Non-ST Segment Elevation Myocardial Infarction; Dysrhy=Dysrhythmia; HTN=Hypertension; AVE=24-hour average; Ave6=6-hour average, AVE12=12-hour average, MAX=maximum 1-hour; MIN=minimum 1-hour. Data were calculated for an inter-quartile range increase of NO2_AVE (15 µg/m^3^), NO2_AVE12 (16 µg/m^3^), NO2_AVE6 (18 µg/m^3^), NO2_MIN (9 µg/m^3^), NO2_MAX (21 µg/m^3^), O3_MIN (7 µg/m^3^), O3_MAX (17 µg/m^3^), PM25_AVE12 (8.3 µg/m^3^), PM25_MIN (3.1 µg/m^3^).**

**^a^ Median value from 1,000 model replications.**

**^b^ Number of times that a variable was significant (p-value ≤0.05) from 1,000 model replications.**
